# When foods become remedies in ancient Greece: The curious case of garlic and other substances

**DOI:** 10.1016/j.jep.2014.08.018

**Published:** 2015-06-05

**Authors:** Laurence Totelin

**Affiliations:** School of History, Archaeology and Religion, Cardiff University, Colum Drive, Cardiff CF10 3EU, United Kingdom

**Keywords:** History, Food-drug continuum, Hippocratic corpus, Dietetics, Garlic, Silphium

## Abstract

**Ethnopharmacological relevance:**

The debate on the food-drug continuum could benefit from a historical dimension. This study aims at showing this through one case: the food-drug continuum in Greece in the fifth- and fourth-century BCE. I suggest that at the time the boundary between food and drug – and that between dietetics and pharmacology – was rather blurred.

**Materials and methods:**

I study definitions of ‘food’ and ‘medicine’ in texts from the fifth- and fourth-century BCE: the Hippocratic texts, the botanical treatises of Theophrastus and the pseudo-Aristotelian *Problems*. To illustrate these abstract definitions, I focus on two substances: garlic and silphium.

**Results and discussion:**

The Hippocratics were writing in a context of increased professionalization and masculinization of medicine, a context in which dietetics became the most prestigious branch of medicine, praised above pharmacology and surgery. While medicine was becoming more specialised, professionalised and masculine, it avoided becoming too conspicuously so. The Hippocratic authors sometimes noted that medical discoveries are serendipitous and can be made by anyone, whether medically trained or not. By doing so, they allowed themselves to integrate common knowledge and practice into their writings.

**Conclusion:**

In the context of the professionalization of ancient medicine, the Hippocratic authors started to address the difference between food and medicine. They saw, however, some advantage in acknowledging the continuum between food and medicine. Scholars should avoid drawing too strict a boundary between ancient dietetics and pharmacology and should instead adopt a multi-disciplinary approach to the therapeutics of the Hippocratic texts.

## Graphical abstract

**Figure:**
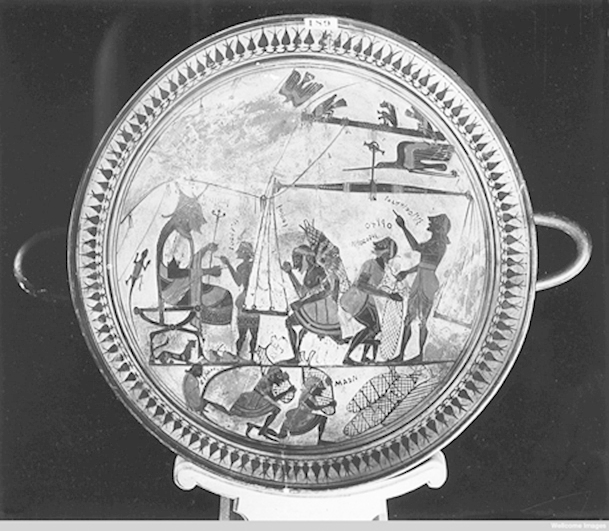

## 1 Introduction

In his *Memorabilia*, Xenophon (c. 430–354 BCE), one of the students of Socrates reports the following dialogue between the philosopher and one of his interlocutors, Euthydemus, on the topic of deception:

‘Suppose then,’ Socrates said, ‘that a general, seeing that his army is in low spirits, tells them a lie and says that allies are approaching, and through that lie, checks the despondency among his soldiers. On which side shall we put this deception?’

‘It seems to me,’ I said, ‘to be on the side of justice.’

‘Suppose now that a man, when his son is in need of drugs (*pharmakeia*), but refuses to take his medicament (*pharmakon*), deceives him by giving that medicament (*pharmakon*) as if it were a food (*sition*), and through this lie restores him to health, where shall we put this deception?’

‘It seems to me,’ I said, ‘that it also goes on the same side.’ [Xenophon, *Memorabilia* 4.2.17; all translations from the Greek and Latin are my own]

Socrates here distinguishes between two categories: that of drug/medicament (*pharmakon*) and that of food (*sition*), indicating that one can easily be dissimulated as the other. The question of the continuum between food and medicine is one that is of great interest to ethnopharmacologists (Etkin and Ross, 1982, 1991; Johns, 1990; Etkin, 2008; Leonti, 2012; Valussi and Scirè, 2012). Here I wish to add a historical dimension to these ethnopharmacological works. In this paper, I attempt to understand how the medical authors active at the same time as Socrates and his students, the Hippocratic authors, conceived of the difference between food and drug. I argue that they deliberately avoided distinguishing too systematically between the two categories in order to account for some versatile substances. To illustrate this, I will use the examples of garlic, which today too poses classificatory problems, and silphium, a plant that is now extinct. My enquiry will allow me to touch on some epistemological issues relating to the perceived superiority of ancient dietetics over pharmacology.

## 2 Materials and methods

My primary source materials will be the collection of earliest medical texts written in Greek: the Hippocratic Corpus (Jouanna, 1999; see Nutton, 2013 for a general introduction to ancient medicine). This is a heterogeneous compilation of some sixty medical texts, written for the most part at the end of the fifth century BCE and in the fourth century BCE, although some texts are significantly later. The name and authority of Hippocrates, the father of medicine, was bestowed upon the compilation, but it is certain that Hippocrates himself could not have composed all the treatises of the collection. Indeed, there are numerous style and content discrepancies to be observed within the collection. For this reason, I will refer to ‘Hippocratic authors’ rather than to Hippocrates. In addition to the Hippocratic Corpus, I will also make reference to contemporary authors such as the comedian Aristophanes (c. 446 BCE–c. 386 BCE), the philosopher and botanist Theophrastus (c. 371–287 BCE) and Pseudo-Aristotle. My method is in the main historical – it consists in analysing in depth ancient texts – but it is informed by gender studies and anthropology. In particular, I will call upon the work of anthropologists who have worked on the question of the continuum between food and medicine. Nina Etkin and Paul Ross, two pioneers in that field, noted that one should use ‘a multi-contextual framework for assessing the physiologic import of plant utilization, to help to move the field beyond the contriving of simple, abstracted catalogues of constituents and activities to the assessment of interdependent uses of plants by real populations in specific cultural contexts׳ (Etkin and Ross, 1982: 25). History, one could argue, has an important role to play in this area of research, because historical texts (in particular herbals) usually give a theoretical framework for the understanding of the difference between food and medicine. While ethnopharmacologists have turned their attention to historical herbals for bioprospecting (e.g. Riddle, 1987; Holland, 1994; Riddle, 2002; Buenz et al., 2004; Lardos et al., 2011) or to stress continuity between ancient and current practises (Pollio et al., 2008; Leonti et al., 2009; De Vos, 2010; Leonti et al., 2010; Lardos and Heinrich, 2013), there still is much work to be done on those theoretical frameworks. It is important that this aspect of ancient pharmacological systems not be ‘lost in translation’, as it were (on the links between ethnobotany and historical sciences, see Heinrich et al., 2006). Historians can also shed light on processes of pharmacological knowledge transmission in ancient cultures, in particular on the questions of the interplay between orality and literacy (Leonti, 2011; Totelin, 2009); between lay and professional medical practitioners; and between men and women in this transmission.

### 2.1 Theory

The theory I want to test here is that the boundary between ‘food’ and ‘drug’, and hence that between dietetics and pharmacology, was left deliberately blurred in the earliest medical texts written in Greek. I do not mean to say that the Greeks did not have a clear vocabulary to refer to ‘foods’ (*sitia*), nourishment (*trophē*), and drugs (*pharmaka*) – as we saw in the text of Xenophon, they did. In this respect they are different from the Hausa of Nigeria whose word *magani* covers both ‘plants administered to cure fever’ and ‘foods used to remedy hunger’ (Etkin and Ross, 1991: 25). While no Hippocratic author ever wrote ‘let food be your medicine, let medicine be your food’ (this saying is often repeated in scholarship: see e.g. Etkin, 2008: 2; Leonti, 2012: 1), they often referred to both drugs and foods in their descriptions of treatments, as in the following example:

In this case [a black disease] it is necessary to purge with medicaments (*pharmaka*) [that purge] from below and from above, and after that to drink ass’s milk, and use foods (*sitia*) that are as emollient and cold as possible: shore-fish, cartilaginous fish, beet, colocynth, and minced meat. [Hippocratic Corpus, *Diseases* 2.74, edition: Jouanna, 1983: 213–214].

As is the case here, in the Hippocratic Corpus, the word ‘*pharmakon*’ usually refers to a purgative drug (laxative or emetic) and the word ‘*sition*’ refers to solid items of food (Artelt, 1968; Goltz, 1974; Lonie, 1977). Normally Hippocratic physicians proscribed the use of solid foods until a disease had reached a ‘crisis’, a turning point. The Corpus contains two catalogues of foods: one in an appendix to *Regimen in Acute Diseases* (chapter 68; edition: Joly, 1972: 89–90), the other in the second book of *Regimen* II (chapters 39–56; edition: Jones, 1931: 306–343; on the catalogue see Wilkins, 2004). On the other hand, the Hippocratic Corpus does not contain catalogues of what would later be called ‘simple drugs’, catalogues of ingredients and their properties. In fact the Hippocratic Corpus does not have any treatise that is devoted entirely to pharmacology, although it does refer to *Pharmakitides*, recipe books that have now been lost. These appear to have contained both what we would classify as pharmacological recipes and dietetic prescriptions, including recommendations relating to *sitia* (Schöne, 1920; Monfort, 2002; Craik, 2006: 17; Totelin, 2009: 98–102). The Corpus also contains a large number of what we would term pharmacological recipes, most of which are to be found in the gynaecological texts (Stannard, 1961; Goltz, 1974; Scarborough, 1983; Hanson, 1991, 1992, 1998, 1999; King, 1995a, 1995b, 1998; Laskaris, 1999; Totelin, 2009).

Thus, the Hippocratic authors had a clear vocabulary to refer to foods and drugs. On the other hand, they avoided defining the difference between the two categories. The closest one comes to such a definition is this passage in the Hippocratic treatise *Places in Man* (which probably dates to the fifth century BCE):

All things that cause change in the present state are drugs (*pharmaka*), and all substances that are rather strong cause change. It is possible, if you want, to bring about change by means of a drug (*pharmakon*), or, if you do not want [to use a drug], by means of food (*sition*). [Hippocratic Corpus, *Places in Man* 45, edition: Craik, 1998: 82].

‘Food’ is something that is unlike ‘drug’, and a drug is something that is rather strong and can therefore effect change. Compare this Hippocratic near-definition to the following systematic discussion, which is to be found in the Aristotelian *Problemata*, a large collection of problems presented in a question-and-answers format, and circulated under the name of Aristotle (Touwaide, 1996; Mayhew, 2013). The question under observation is ‘why is it that not all purgative drugs are bitter in taste’:

Oil and honey and milk and other such nourishment (*trophē*) purge, but it depends not on their kind but on their quantity. For if they are to purge, it is only when, on account of quantity they are uncocted, that they do so. Substances are uncocted for two reasons: either because of quality or because of quantity. This is why none of the substances mentioned above are drugs (*pharmakon*). For they do not purge on account of their properties. Astringency, bitterness and foul smell are characteristics of drugs (*pharmaka*) because a drug (*pharmakon*) is the opposite of nourishment (*trophē*). For what is concocted by nature causes bodies to grow and is called nourishment (*trophē*). But that which by nature cannot be overcome enters into the veins and, because of an excess of heat or cold causes disturbance, that is of the nature of a drug (*pharmakon*) [Aristotle, *Problemata* 1.42, 864b; edition: Mayhew, 2011: 40–42].

This definition centres on the Aristotelian notion of coction: a food can be overcome (cooked) by the body, whereas a drug cannot. A food taken in too large a quantity can have the action of a drug, because the body will not be able to digest it all. There are two issues with the pseudo-Aristotelian definition. First, it is nigh-on impossible to date. Even though some of the so-called ‘Aristotelian’ problems may have originated in the Aristotelian school, and include Aristotelian notions such as that of coction, they seem to have evolved with time and crystallised in the form that we know only quite late in antiquity. In other words the question ‘what is the difference between a food and a drug’ may have been asked at the time of Aristotle (and well before), but may not have received such a clear answer at that point. The second problem is that this definition has a rather restrictive notion of *pharmakon*: the *pharmakon* causes a purge and has to be ingested by mouth. What about drugs applied externally and those that have an action different from purging?

These difficulties aside, the pseudo-Aristotelian discussion is interesting from an ethnopharmacological point of view, as it establishes a link between ‘bitterness’ and ‘drug’ (see Johns, 1990). The philosopher Theophrastus, successor of Aristotle at the head of the Lyceum, also observed that link: sweet things tend to be more nutritive than bitter ones; they lend themselves better to being used as foods. Bitterness, on the other hand, is often a characteristic of medicinal plants. The matter was, however, more complicated:

Not all sweet flavours are nutritious for us: some cause mental derangement, like the root that is similar to golden thistle and some other roots; some are soporific and, when given in large amounts, are even lethal, like mandrake; and some are admittedly deadly. For many, in many places, have eaten roots which they perceived to be sweet to the taste and pleasant, and died as a result. And many other plants that harm or even kill are sweet or cause no pain when they are first ingested. Then again, some unpleasant and bitter plants are beneficial, as the centaury and wormwood just mentioned, and some others which taste even more drug-like (*pharmakōdesterous*) are also good for us. (Theophrastus, *Causes of Plants* 6.4.5-6; edition: Einarson and Link, 1990b: 244-246)

Thus while Aristotelian philosophers attempted to define the difference between ‘food’ and ‘drug’, the Hippocratics were avoiding to do so. I would argue they did this because any definition would be, like the Aristotelian one, reductive. To illustrate this, I will now discuss two concrete examples: garlic (Greek *skorodon*: the Greek word may at times have been used to designate other plants that were similar to, but not *Allium sativum* L., family: Amaryllidaceae) and silphium (see below for identification). These are two examples among many, which I have chosen for specific reasons. Garlic was a staple in ancient Greece, as it is today. It would be very tempting to look for continuity in use, but this is not quite substantiated by our ancient evidence. Silphium, for its part, was a plant that had a limited geographical distribution and that was extremely expensive. It is now believed to be extinct. It forces us to consider the food-drug continuum in a historical context, while also stressing the possible environmental impact of extensive plant exploitation.

Today, garlic is primarily an item of diet, used to flavour dishes. However, there also are a plethora of garlic supplements, which claim to have a positive effect on the cardio-vascular system – they are believed to lower cholesterol, even though scientific experiments are not that conclusive. Leonti et al. (2010: 389) have shown that no ancient Western medical text (Hippocrates, Galen, Hildegard von Bingen etc.) mention cardio-vascular properties for garlic. These were discovered in the mid-twentieth century. Garlic has since acquired the status of ‘traditional’ remedy for hypertension and high cholesterol, when we are actually dealing with the rapid diffusion of scientific biomedical knowledge within the larger community. Other possible properties of garlic are also being investigated; recent laboratory tests have shown that garlic has antibacterial, antiviral and antifungal properties. Research is ongoing as to the possible medical uses of garlic and its extracts. This is a clear example of a food being used in health contexts (see e.g. Gardner et al., 2007; Reinhart et al., 2009; Tsai et al., 2012; Khatua et al., 2013; Ried et al., 2013).

In the ancient world, garlic was primarily an item of diet, much derided by comic authors because of its well-known impact on the breath (Wilkins, 2000). It was consumed in such quantities that during the Hellenistic period (the period after the death of Alexander the Great in 323 BCE), there were some attempts at specialised cultivation of garlic in Ptolemaic Egypt (Crawford, 1973). In addition to being an item in everyday diet, garlic was often recommended by medical authors as a food to be used by patients following a treatment. For instance, the compiler of the Hippocratic treatise *Nature of Woman* advised a woman treated for ‘displacement of the womb’ through purgatives, fumigations, manipulations and other applications, to take the following foods:

And let her eat a lot of garlic, both raw and boiled, and drink its juice as a soup, and let her make use of emollient foods [Hippocratic Corpus, *Nature of Woman* 6; edition: Potter, 2012: 201]

At times, however, garlic enters lists of foods a patient must avoid, for reasons that are not always easy to understand, but mostly because it was believed to be a windy plant, a plant that causes flatulence. In the catalogue of foods in *Regimen* II, we find a more theoretical exposition of the properties of garlic as an item of food:

Garlic is warm, excretive and diuretic. It is excretive and diuretic because of its purgative quality. It is good for the body though bad for the eyes. For by making a considerable purgation of the body it dulls the sight. When boiled it is weaker than when raw. It creates flatulence because it stops the *pneuma* [i.e. a vital breath]. [Hippocratic Corpus, *Regimen* 2.54.1; edition: Joly, 1972: 51-52].

Not all these claims seem to have been accepted by all Hippocratic physicians, however. In particular, the claims relating to the eyes seem to have been disputed. The author of *Organ of Sight* recommends eating raw garlic in the treatment of night blindness (chapter 7; edition Craik, 2006: 44); and that of *Epidemics* 2 prescribes eating garlic and barley cake after the application of an eye remedy (*Epidemics* 2.5.22; edition Smith, 1994: 78). In later medical works, one reads of remedies containing garlic to be applied directly to the eyelids (e.g. Pseudo-Galen, *Remedies Easily Prepared* 1.14; edition Kühn vol. 14, p. 343). One does not find a remedy of this type in the Hippocratic Corpus, but a contemporary of the Hippocratic writers, the comedian Aristophanes, creates a comic remedy in which garlic has to be applied to the inside of the eyelids:

First of all, for Neocleides, he [Asclepius, the God of Medicine] set himself to knead a plaster, throwing in three cloves of Tenian garlic. Then, he crushed them in the mortar, mixing them together with verjuice and mastic. Then, he soaked the mixture with Sphettian vinegar. And turning out the eyelids of the man, he plastered them to make him suffer more. [*Plutus* 716–722]

With these uses of garlic in the treatment of eye diseases, we have imperceptibly slipped into a discussion of garlic as a drug rather than a food. Another non-dietetic use of garlic in the classical period was in fertility tests:

Another: Having washed and peeled a head of garlic, apply it to the womb, and see the next day whether she smells of it through the mouth; if she smells, she will be pregnant, if not, she will not [Hippocratic Corpus *Barren Women* 214; edition Potter, 2012: 338-340]

The ancients imagined that women had a sort of tube in their body, leading from the vagina to the mouth. That tube could easily get blocked, thus preventing conception. A test like this one would determine whether there was a blockage: if the smell of the garlic travels from the vagina to the mouth, there is none and the woman will conceive (de Crusance Morant Saunders, 1963; Iversen, 1939; Jouanna, 2004; Marganne, 1993; Totelin, 2009: 181–183).

In the context of sexuality, it is also interesting to note that women participating in the women-only Athenian festival *Skira* consumed large quantities of garlic. The explanation given for this practice is that the smell was supposed to keep husbands away; in the words of the historian Philochorus (third century BCE), ‘they ate garlic in order to abstain from sex, so that they would not smell of perfume’ (FGRH 328 F 89; see Parker, 2005: 174).

Finally, we find references to garlic in recipes ‘to create a wind in the womb’: ‘If you want to create a wind in the womb: add to pessaries a head of garlic and the juice of silphium.’ (Hippocratic Corpus, *Barren Women* 239; edition Potter, 2012: 382). ‘Creating a wind in the womb’ through the use of windy plants such as garlic may have allowed to remove blockages preventing conception; it may also have sometimes induced an abortion (Riddle, 1997: 44). In favour of this interpretation, it can be noted that the second ingredient of the recipe, silphium, occurs in recipes to expel a dead foetus (Hippocratic Corpus, *Diseases of Women* 1.91; edition: Littré, vol. 8, p. 218).

This ‘windy’ recipe introduces my second example of a plant that can be classified either as a food or as a drug: silphium. It was a plant from Cyrene (a Greek city-state in northern Africa); growing only in the wild and with a limited geographical distribution. The Greeks had attempted to grow it in the Peloponnese and in Ionia, but had failed (*Diseases* 4.34; edition Potter, 2012: 104). Theophrastus of Eresus mentions silphium among those plants that are ‘emasculated’ (*ekthēlunetai*) by cultivation: ‘for it does not have the same pungency [when cultivated] because its nourishment is too abundant and watery’ (*Causes of Plants* 3.1.3 and 5; edition: Einarson and Link, 1990a: 4–8). There was no choice but to import it at great cost from Cyrene (Aristophanes, *Plutus* 923–925). In the first century CE, the plant was reported to be extinct. The Roman encyclopaedist Pliny the Elder (23–79 CE) wrote that:

It has not been found in this land [Cyrene] for many years, because the tax-farmers who rent the pasture-land destroy it by grazing sheep on it, believing that they would make more profit this way. There has only been one stem found in living memory; it was sent to the Emperor Nero [*Natural History* 19.39; edition: Rackham, 1950: 444].

This explanation is spurious for two reasons. First, the price of silphium is always reported as high; how could tax-farmers have believed they could make more profit by using the land for pasture? Second, we find references to silphium in texts well beyond the first century CE. This second point can be explained in two ways: either the plant was not really extinct or people were now using a replacement plant: the silphium from Media or Persia, identified with our asafoetida (*Ferula asafoetida* L.) (on the possible extinction of silphium, see: Andrews, 1941; Parejko, 2003; Roques, 1984). Indeed thanks to descriptions in ancient texts and representations on Cyrenaic coins, we can identify silphium with an umbellifer quite similar to *Ferula asafoetida* L. (family: Apiaceae). The real identity of silphium, however, remains a mystery. Table 1 shows the identifications that have been offered for the plant (for fuller discussion see Amigues, 2004).

Recent studies on silphium’s ancient medical properties have focused on its use as a contraceptive/abortive (Riddle, 1991, 1992, 1997) or as an aphrodisiac (although scholars who have advanced this hypothesis recognise it is not substantiated in ancient texts; Koerper and Kolls, 1999; Koerper and Moerman, 2000). There were other uses of the plant, however. In the Hippocratic Corpus, it is listed in the catalogue of foods in *Regimen in Acute Diseases* (chapter 68; edition Joly, 1972: 89–90), and it is recommended as a food in ‘windy diets’. The following example is part of an extremely long treatment for the ‘displacement of womb to the hip-joint’ (on ‘displacements of the womb’, see King, 1998), which involves purges, fumigations, pessaries and baths, and also outlines the diet of the patient:

Make her eat barley-cake or bread, and leeks cooked or raw, and all similarly sharp things prescribed above, and now and then, thick soups from which the foam has not been removed, and a lot of crushed silphium and quantity of boiled garlic. [Hippocratic Corpus, *Diseases of Women* 2.133; edition: Littré, vol. 8, p.298]

The meal described here is not particularly different from a staple Greek meal, although silphium, unlike garlic, was too expensive to be consumed on a regular basis. One could also mention the following dish, prescribed in the case of a ‘typhus’, which incidentally has been caused by an excess of food (*sitia*) and in particular of seasonal fruits (*opōrē*):

After the purge with the juice [of lentils], in the evening, let the patient sup on a bowl of cold, unsalted lentil-soup, on which much silphium has been grated. [Hippocratic Corpus, *Internal Affections* 42; edition: Potter, 1988b: 212]

Again, lentil soup was a staple meal in the ancient world, although it would probably have been seasoned with salt rather than with expensive silphium. In their study of Hausa therapy, Etkin and Ross have noted that pharmacological plants were often ‘added to foods in dishes prepared for the ailing individual(s) and not for all members of the household unit who ordinarily “eat from the same pot” (Etkin and Ross, 1982: 1560). In our ancient example, it is conceivable that the lentil soup was prepared for the entire household, but the silphium reserved for the sick.

## 3 Results

In both our examples, I would argue, the ancients observed the properties of plants that were used primarily as foods, and then applied these properties medicinally. Both garlic and silphium were windy plants, whose smell could travel through the body. They could cause flatulence, but used in large amounts, they could also create enough wind to remove blockages in the body. In addition to its windiness, garlic also made the eye cry, hence its use in ophthalmology. In any case, the medicinal uses of garlic and silphium stem from observations made in cooking. There is a continuum between cooking and medicine, that is, ancient cooking and medicine share the same processes and understanding of substances’ effect on the body. The definition of the Aristotelian *Problemata*, which made a clear distinction between food and drug, simply does not work in the cases of garlic and silphium, not the least because in some cases these plants were not ingested but applied externally. The professional-looking neatness of this definition does not encompass the messy reality of health-giving substances such as garlic and silphium.

## 4 Discussion

The Hippocratic authors wrote at a time of what we can call increased professionalization. Various ‘professions’, including the medical one, were attempting to establish their legitimacy and define the boundaries of their *technē*, their art (von Staden, 1996). In medicine, this would eventually lead to the neat division of therapeutics into three branches, as expressed in the preface to Celsus’ (a Roman encycolapedist active in the first century CE) *On Medicine*:

During the same times the Art of Medicine was divided into three parts: one being that which cures through diet, another through medicaments, and the third by hand. The Greeks termed the first *diaitētikē* (dietetics), the second *pharmakeutikē* (pharmacology), the third *cheirourgia* (surgery). [Celsus, *On Medicine*, preface 9].

That neat division is not quite present in the Hippocratic treatises themselves, although a loose one is to be observed in some parts of the Corpus (Goltz, 1974: 297–302; Lonie, 1977: 245; von Staden, 1999: 257-258; Thivel, 2000: 35–37; Holmes, 2010: note 161). Particularly blurred was the boundary between pharmacology and dietetics, the Greek branch of medicine that embraced most aspects of daily life: diet, obviously, but also exercises, baths, even sexual intercourse – whether in sickness or in health (Scarborough, 1970; Ackerknecht, 1973; Lonie, 1977; Smith, 1980; Scarborough, 1982; Edelstein, 1987; Mazzini, 1989; Sigerist, 1989; Jori, 1993; Craik, 1995a, 1995b; King, 1995a, 1995b; Longrigg, 1999; Steger, 2004). The Greeks considered dietetics to be a sixth- or fifth-century BCE invention, even though they could not agree on the name of its inventor: was it the philosopher Pythagoras of Samos; the athlete turned physician Herodicus of Selymbria; or the physician Hippocrates of Cos (Longrigg, 1998: 147–148; Smith, 1999; Thivel, 1999)?

In any case, at the end of the fifth century BCE, dietetics had become an established discipline, which the author of the Hippocratic treatise *On Ancient Medicine* equated with medicine itself:

For the art of medicine would never have been discovered to begin with, nor would any medical research have been conducted – for there would have been no need for medicine – if sick men had profited by the same mode of living and regimen as the food, drink and mode of living of men in health, and if there had been no other things for the sick better than these. But the fact is that sheer necessity has caused men to seek and to find medicine, because sick men did not, and do not, profit by the same regimen as do men in health [Hippocratic Corpus, *Ancient Medicine* 3; edition Jouanna, 1990: 120-121].

Dietetics would in later antiquity become the most prestigious therapeutic branch. In Celsus’ work for instance, it was the branch that deserved to be treated first and at most length. Many modern scholars follow Celsus in seeing ancient dietetics as superior to pharmacology or surgery. For instance, Michel Foucault (1984: 21) argued that the body is passive when undergoing pharmacological or surgical treatments, while the soul is actively learning when dietetic treatment is used (see also Temkin, 1953: 221; Sassi, 2001: 142). Such views have meant that, until the 1980s, the gynaecological treatises of the Hippocratic Corpus, which contain many pharmacological recipes, were neglected by scholars. I would argue, however, that these views are misleading and that there was a strong continuum between dietetics and pharmacology in the ancient world.

By regulating all aspects of people׳s life through dietetics, the Hippocratics would have certainly stepped on the territory of other people, such as professional cooks, or women, who were mostly responsible for food preparation in the Greek world. The physicians cleverly avoided that accusation by acknowledging the role of self-treatment (King, 1995a, 1995b), and by claiming that anyone could contribute to the advancement of medical knowledge:

It is worth learning from everyone about drugs (*pharmaka*) that are drunk or applied to wounds. Indeed men do not discover these by reasoning, but rather by chance, and not more by experts (*cheirotechnai*) than by laymen (*idiōtai*). But whatever is discovered in the art of medicine by reasoning, whether about foods (*sita*) or about drugs (*pharmaka*), must be learnt from those who have discernment in the art of medicine, if you want to learn anything. [Hippocratic Corpus, *Affections* 45; edition Potter, 1988a: 68].

The Hippocratic authors were quite rightly aware of the serendipitous nature of much medical discovery, and did not want to miss opportunities to find new treatments coming from unlikely sources, or indeed to appropriate for themselves common practice located in the household. While knowledge of clearly medicinal plants such as hellebore or mandrake was limited to herbalists and physicians (physicians: *iatroi*; root cutters: *rhizotomoi*; drug-sellers: *pharmakopolai*; ‘witches’: *pharmakides*), that of staple plants was diffused through society. Among the laymen that contributed to discoveries in the fields of dietetics and pharmacology, there were women. The anthropologist Jack Goody has noted that increased specialization and professionalization often leads to a ‘sexual transposition of domestic tasks’, that is, tasks that would have been performed by women are appropriated by ‘professionals’, who usually are male (Goody, 1982: 101; see also Garnsey, 1999: chapter 8). That is not to say that there were no female practitioners in the ancient world – there were midwives (*maiai*); female midwife-doctors (*iatromaiai*); and female doctors (*iatrinai*) (see e.g. Parker, 1997; Flemming, 2007; Muir and Totelin, 2012) – but they are mentioned far less often than men in the ancient literature, and are often presented in a negative light. Ancient literature gives us a sense that the ‘medical market-place’ was dominated by men (Nutton, 1992). Since the 1980s, however, feminist historians, have argued that the involvement of women may have been much more significant than ancient texts lead us to believe. This argument has focused on the gynaecological – pharmacological – recipes of the Hippocratic Corpus (Rousselle, 1980; McLaren, 1990: 28; Hanson, 1991, 1992, 1998, 1999; Riddle, 1992; Demand, 1994; Dean-Jones, 1994; King, 1995a, 1995b, 1998; Totelin, 2009). The same debate has not yet happened in relation to Greek dietetics more generally. In other words, while it is now common among historians to discuss the involvement of women in ancient pharmacology, it is not yet the case for ancient dietetics.

## 5 Conclusions

In this paper, I have suggested that at the time of the Hippocratic writers, the boundary between food and drug, pharmacology and dietetics, was still rather blurred. The Hippocratics knew that some everyday foods could bring important health benefits and could be used in non-dietetic ways, and for that reason they may have wished not to come up with too systematic a definition of the boundary between foods and drugs. The Hippocratics also knew that laymen and laywomen could contribute discoveries to the field of medicine, and in that matter also not too strict a definition of the boundary between food and drug may have helped them. However, the Hippocratic writers were also keen to establish their authority and distinguish themselves from those laymen and laywomen. This would eventually lead to the writing down of systematic, theoretical definitions of the difference between food of drug; definitions that can never encompass the complex nature of organic substances.

Of course, in practice, substances such as garlic were not affected by the increasing professionalization in Greek medicine – they could still be used both as foods and drugs. And, garlic is in fact described both in pharmacological and dietetic treatises in later antiquity. The difference was more in attitudes: the newly-defined dietetics would become the most prestigious branch of medicine in the ancient world.

While scholars are aware of the existence of female practitioners in the ancient world, I would suggest that they have internalized a model whereby inferior pharmacology has been associated with women and superior dietetics has been associated with males.

This internalization contributed for many years to a neglect of the Hippocratic gynaecological treatises and the recipes contained therein. This situation has now been remedied, but a feminist approach to other parts of the Corpus, and in particular to the food-related knowledge it contains is still lacking. I hope this paper, which claims that the boundary between food and drug was particularly fluid in the early days of written Greek medicine, contributes in a small way to redressing the balance and will invite further discussion on the possible involvement of women in the development of ancient dietetics, pharmacology and medicine in general, as well as on the ‘ownership’ of herbal knowledge in the modern medical market place.

## Figures and Tables

**Table 1:** Possible identifications for silphium.

Identification proposed by	Year when the identification was proposed	Identification proposed	Reference
Kurt P.J. Sprengel	1807	*Ferula tingitana* L.	Sprengel, 1807: 39–40
Paolo Della-Cella and Domenico Viviani	1817	*Thapsia silphium*⁎	Della Cella and Viviani, 1819; Cauvet, 1875: 13
Heinrich Link	1818	*Laserpitium gummiferum* Desf.=*Margotia gummifera* (Desf.) Lange	Schneider, 1818: 483
Ørsted	1869	*Ferula narthex* Boiss.	Ørsted, 1869
M. Laval	1874	*Thapsia garganica* L.	Cauvet, 1875: 11
M.A.T. Vercoutre	1908	*Lodoicea Sechellarum* Labill.	Vercoutre, 1913
A.Manuta	1996	*Cachrys ferulacea* L.	Manunta, 1996

⁎ No author citation was given for the name *Thapsia silphium* in the original literature.
